# 6,7-Bis(bromo­meth­yl)-2,11,18,21,24,27-hexa­oxatetra­cyclo­[26.4.0.0^4,9^.0^12,17^]dotriaconta-1(28),4,6,8,12(17),13,15,29,31-nona­ene dichloro­methane monosolvate

**DOI:** 10.1107/S160053681200565X

**Published:** 2012-02-17

**Authors:** Hyun Jin Beack, Seung Mee Yoo, Jae Eun Kim, Wonbo Sim, Jai Young Lee

**Affiliations:** a Department of Chemistry, Konyang University, Nonsan 320-711, Republic of Korea

## Abstract

The title 20-crown-6 unit, C_28_H_30_Br_2_O_6_·CH_2_Cl_2_, consisting of three benzo groups and triethyl­ene glycol was prepared from the reaction of 1,2,4,5-tetra­kis­(bromo­meth­yl)benzene and bis­phenol in the presence of sodium hydride. In the crystal, one O atom of the central ethyl­ene glycol in the triethyl­ene glycol unit exhibits an *exo* conformation as a result of intra­molecular C—H⋯O hydrogen bonds. The crown unit and the solvent mol­ecule are linked by weak C—H⋯O hydrogen bonds.

## Related literature

For background to crown ether-based inclusion behaviour, see: Wolf *et al.* (1987 ▶). For the preparation and crystal structures of related compounds, see: Sim *et al.* (2001 ▶); Lee *et al.* (2009 ▶).
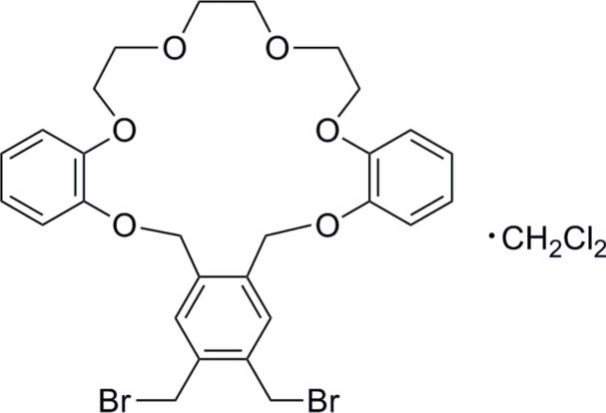


## Experimental

### 

#### Crystal data

C_28_H_30_Br_2_O_6_·CH_2_Cl_2_
*M* *_r_* = 707.27
Triclinic, 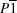

*a* = 9.133 (3) Å
*b* = 12.191 (4) Å
*c* = 15.038 (6) Å
α = 66.116 (7)°
β = 85.263 (9)°
γ = 77.080 (6)°
*V* = 1492.1 (8) Å^3^
*Z* = 2
Mo *K*α radiation
μ = 2.94 mm^−1^
*T* = 200 K
0.35 × 0.16 × 0.14 mm

#### Data collection

Bruker SMART CCD area-detector diffractometer
Absorption correction: multi-scan (*SADABS*; Sheldrick, 1996 ▶) *T*_min_ = 0.575, *T*_max_ = 0.663
11188 measured reflections
7310 independent reflections
3257 reflections with *I* > 2σ(*I*)
*R*_int_ = 0.050

#### Refinement

*R*[*F* ^2^ > 2σ(*F* ^2^)] = 0.079
*wR*(*F* ^2^) = 0.243
*S* = 1.05
7310 reflections
352 parameters
H-atom parameters constrained
Δρ_max_ = 0.96 e Å^−3^
Δρ_min_ = −0.99 e Å^−3^

### 

Data collection: *SMART* (Bruker, 2000 ▶); cell refinement: *SAINT-Plus* (Bruker, 2000 ▶); data reduction: *SAINT-Plus* program(s) used to solve structure: *SHELXTL* (Sheldrick, 2008 ▶); program(s) used to refine structure: *SHELXTL*; molecular graphics: *SHELXTL*; software used to prepare material for publication: *SHELXTL*.

## Supplementary Material

Crystal structure: contains datablock(s) I, global. DOI: 10.1107/S160053681200565X/lx2226sup1.cif

Structure factors: contains datablock(s) I. DOI: 10.1107/S160053681200565X/lx2226Isup2.hkl

Supplementary material file. DOI: 10.1107/S160053681200565X/lx2226Isup3.cml

Additional supplementary materials:  crystallographic information; 3D view; checkCIF report

## Figures and Tables

**Table 1:** Hydrogen-bond geometry (Å, °)

*D*—H⋯*A*	*D*—H	H⋯*A*	*D*⋯*A*	*D*—H⋯*A*
C15—H15*B*⋯O5	0.99	2.38	2.996 (9)	120
C29—H29*B*⋯O5	0.99	2.42	3.352 (13)	158

**Fig. 1.**
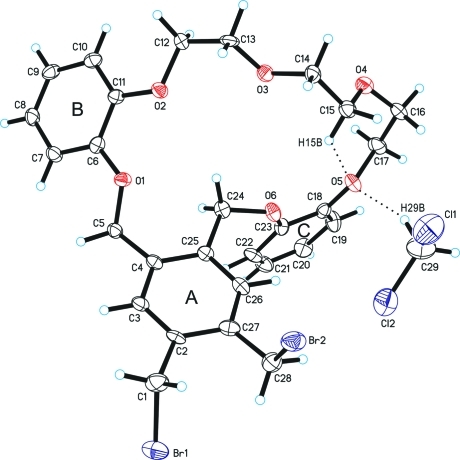
The molecular structure of the title compound with the atom numbering scheme and C–H···O interactions (dotted lines). Displacement ellipsoids are drawn at the 30% probability level. H atoms are presented as small spheres of arbitrary radius.

## supplementary crystallographic information

### Comment

In our previous paper (Sim *et al.*, 2001, Lee *et al.*, 2009), we reported the preparation of new crown ether and its solid-state structure, which could be a precursor of the common-nuclear biscrown ether, bearing three aromatic subunits. Herein, we report the crystal structure of the title compound.

In the title molecule (Fig. 1), in the A-to-B ring and A-to-C ring connectivities, the torsion angles C4–C5–O1–C6 and C25–C24–O6–C23 are 169.4 (5)° and 101.4 (7)°, respectively, which indicate that the A ring is situated *trans* to B ring, but situated *gauche* to C ring, with dihedral angles of 41.7 (2)° between A and B and 85.5 (2)° between A and C. The dihedral angle between B and C rings is 78.2 (2)°. The all C–C–O–C torsion angles except C17–C16–O4–C15(87.4 (8)°) in the triethylene glycol group exhibit *trans* conformation.

In the title compound, O4 atom of two oxygen atoms (O3 and O4) of triethylene glycol group is in an *exo*-orientation, whereas O3 is in an *endo*-orientation. In general, oxygen atoms of ethylene glycol groups in crown ether-based compounds would favor *endo*-orientation (Wolf *et al*., 1987). Exo conformation of the O4 atom is due to the intramolecular C–H···O hydrogen bonds (Fig. 1 & Table 1). In addition, the crown unit and the solvent molecule are linked by weak intermolecular C–H···O hydrogen bonds (Fig. 1 & Table 1).

### Experimental

To a refluxing suspension of sodium hydride (5.50 mmol) in THF under nitrogen was added dropwise a solution of 1,2,4,5-tetrakis(bromomethyl)benzene (2.20 mmol) and 1,8-bis(2-hydroxyphenoxy)-3,6-dioxaoctane (2.00 mmol) in THF over a period of 3 h. The mixture was then refluxed for an additional 24 h. After cooling to room temperature, 10% aqueous hydrochloric acid was added. The solvent was removed under reduced pressure and the residual mixture was extracted with dichloromethane. The organic layer was washed with water, dried over anhydrous magnesium sulfate, and evaporated *in vacuo*. The crude product was chromatographed on a silica-gel column using a mixed solvent of ethyl acetate and *n*-hexane (1:1) as eluent, and recrystallization from dichloromethane/*n*-hexane (1:20, *v*/*v*) gave as a crystalline solid in 49% yield (m.p. 373 K).

### Refinement

All H-atoms were positioned geometrically and refined using a riding model with d(C–H)=0.95 Å, *U*_iso_=1.2*U*_eq_(C) for aromatic and 0.99 Å, *U*_iso_=1.2*U*_eq_(C) for CH_2_ atoms.

### Crystal data

**Table:**

C_28_H_30_Br_2_O_6_·CH_2_Cl_2_	*Z* = 2
*M**_r_* = 707.27	*F*(000) = 716
Triclinic, *P*1	*D*_x_ = 1.574 Mg m^−^^3^
Hall symbol: -P 1	Mo *K*α radiation, λ = 0.71073 Å
*a* = 9.133 (3) Å	Cell parameters from 2021 reflections
*b* = 12.191 (4) Å	θ = 2.3–24.1°
*c* = 15.038 (6) Å	µ = 2.94 mm^−^^1^
α = 66.116 (7)°	*T* = 200 K
β = 85.263 (9)°	Block, colourless
γ = 77.080 (6)°	0.35 × 0.16 × 0.14 mm
*V* = 1492.1 (8) Å^3^	

### Data collection

**Table:**

Bruker SMART CCD area-detector diffractometer	7310 independent reflections
Radiation source: fine-focus sealed tube	3257 reflections with *I* > 2σ(*I*)
Graphite monochromator	*R*_int_ = 0.050
Detector resolution: 10.0 pixels mm^-1^	θ_max_ = 28.3°, θ_min_ = 1.5°
π and ω scans	*h* = −12→11
Absorption correction: multi-scan (*SADABS*; Sheldrick, 1996)	*k* = −16→14
*T*_min_ = 0.575, *T*_max_ = 0.663	*l* = −20→10
11188 measured reflections	

### Refinement

**Table:**

Refinement on *F*^2^	Primary atom site location: structure-invariant direct methods
Least-squares matrix: full	Secondary atom site location: difference Fourier map
*R*[*F*^2^ > 2σ(*F*^2^)] = 0.079	Hydrogen site location: difference Fourier map
*wR*(*F*^2^) = 0.243	H-atom parameters constrained
*S* = 1.05	*w* = 1/[σ^2^(*F*_o_^2^) + (0.0893*P*)^2^ + 2.2498*P*] where *P* = (*F*_o_^2^ + 2*F*_c_^2^)/3
7310 reflections	(Δ/σ)_max_ < 0.001
352 parameters	Δρ_max_ = 0.96 e Å^−^^3^
0 restraints	Δρ_min_ = −0.99 e Å^−^^3^

### Special details

**Table:**

Experimental. IR (KBr pellet): 2919, 1599, 1504, 1254, 1212, 1118, 1050 and 748 cm^-1. 1^H NMR (CDCl_3_): δ 7.59 (s, 2 H, BrCH_2_*Ar*), 6.97-6.87 (m, 8 H, O*Ar*O), 5.20 (s, 4H, ArC*H*_2_O), 4.68 (s, 4 H, ArC*H*_2_Br), 4.13 (t, 4 H, ArOC*H*_2_CH_2_OCH_2_), 3.82 (t, 4 H, ArOCH_2_C*H*_2_OCH_2_) and 3.63 (s, 4 H, ArOCH_2_CH_2_OC*H*_2_).
Geometry. All e.s.d.'s (except the e.s.d. in the dihedral angle between two l.s. planes) are estimated using the full covariance matrix. The cell e.s.d.'s are taken into account individually in the estimation of e.s.d.'s in distances, angles and torsion angles; correlations between e.s.d.'s in cell parameters are only used when they are defined by crystal symmetry. An approximate (isotropic) treatment of cell e.s.d.'s is used for estimating e.s.d.'s involving l.s. planes.
Refinement. Refinement of *F*^2^ against ALL reflections. The weighted *R*-factor *wR* and goodness of fit *S* are based on *F*^2^, conventional *R*-factors *R* are based on *F*, with *F* set to zero for negative *F*^2^. The threshold expression of *F*^2^ > σ(*F*^2^) is used only for calculating *R*-factors(gt) *etc*. and is not relevant to the choice of reflections for refinement. *R*-factors based on *F*^2^ are statistically about twice as large as those based on *F*, and *R*- factors based on ALL data will be even larger.

### Fractional atomic coordinates and isotropic or equivalent isotropic displacement parameters (Å^2^)

**Table:**

	*x*	*y*	*z*	*U*_iso_*/*U*_eq_	
Br1	−0.19294 (12)	1.08922 (9)	0.11382 (8)	0.0750 (4)	
Br2	−0.07519 (10)	0.64380 (8)	0.27325 (7)	0.0536 (3)	
O1	0.4952 (5)	0.8625 (4)	0.3957 (3)	0.0314 (11)	
O2	0.6891 (5)	0.6718 (4)	0.4961 (3)	0.0352 (11)	
O3	0.7455 (6)	0.4547 (4)	0.4600 (3)	0.0374 (12)	
O4	0.8421 (6)	0.3167 (4)	0.3362 (4)	0.0421 (13)	
O5	0.7269 (5)	0.5068 (4)	0.1510 (4)	0.0383 (12)	
O6	0.5641 (5)	0.6725 (4)	0.2045 (4)	0.0352 (12)	
C1	−0.1111 (8)	0.9652 (8)	0.2398 (6)	0.049 (2)	
H1A	−0.1290	1.0022	0.2885	0.059*	
H1B	−0.1654	0.8971	0.2612	0.059*	
C2	0.0548 (7)	0.9141 (6)	0.2369 (5)	0.0313 (16)	
C3	0.1536 (8)	0.9547 (6)	0.2733 (5)	0.0313 (16)	
H3A	0.1156	1.0179	0.2959	0.038*	
C4	0.3080 (7)	0.9074 (6)	0.2788 (5)	0.0286 (15)	
C5	0.4108 (7)	0.9594 (6)	0.3164 (5)	0.0308 (15)	
H5A	0.4793	0.9982	0.2643	0.037*	
H5B	0.3515	1.0226	0.3380	0.037*	
C6	0.6127 (8)	0.8848 (6)	0.4308 (5)	0.0306 (15)	
C7	0.6316 (8)	1.0009 (6)	0.4179 (5)	0.0350 (17)	
H7A	0.5604	1.0721	0.3809	0.042*	
C8	0.7548 (8)	1.0117 (6)	0.4594 (5)	0.0371 (17)	
H8A	0.7679	1.0906	0.4506	0.045*	
C9	0.8566 (8)	0.9105 (7)	0.5124 (5)	0.0379 (18)	
H9A	0.9402	0.9196	0.5404	0.046*	
C10	0.8415 (8)	0.7938 (7)	0.5267 (5)	0.0360 (17)	
H10A	0.9143	0.7237	0.5634	0.043*	
C11	0.7193 (7)	0.7816 (6)	0.4866 (5)	0.0291 (15)	
C12	0.7963 (8)	0.5648 (6)	0.5493 (5)	0.0357 (17)	
H12A	0.8080	0.5596	0.6159	0.043*	
H12B	0.8951	0.5675	0.5169	0.043*	
C13	0.7415 (8)	0.4562 (6)	0.5536 (5)	0.0372 (18)	
H13A	0.8057	0.3799	0.5988	0.045*	
H13B	0.6374	0.4599	0.5783	0.045*	
C14	0.6927 (8)	0.3521 (6)	0.4625 (5)	0.0347 (17)	
H14A	0.5897	0.3542	0.4890	0.042*	
H14B	0.7586	0.2751	0.5054	0.042*	
C15	0.6924 (8)	0.3555 (6)	0.3627 (5)	0.0380 (18)	
H15A	0.6288	0.3007	0.3604	0.046*	
H15B	0.6502	0.4399	0.3163	0.046*	
C16	0.8545 (9)	0.3047 (6)	0.2475 (6)	0.044 (2)	
H16A	0.7658	0.2760	0.2386	0.053*	
H16B	0.9445	0.2408	0.2499	0.053*	
C17	0.8662 (8)	0.4209 (7)	0.1606 (6)	0.046 (2)	
H17A	0.9499	0.4547	0.1700	0.055*	
H17B	0.8856	0.4039	0.1012	0.055*	
C18	0.7255 (7)	0.6274 (6)	0.0888 (5)	0.0325 (16)	
C19	0.8058 (10)	0.6615 (7)	0.0047 (6)	0.049 (2)	
H19A	0.8681	0.6009	−0.0140	0.058*	
C20	0.7959 (10)	0.7854 (7)	−0.0535 (6)	0.050 (2)	
H20A	0.8514	0.8094	−0.1121	0.060*	
C21	0.7059 (9)	0.8734 (7)	−0.0264 (6)	0.046 (2)	
H21A	0.7012	0.9579	−0.0655	0.056*	
C22	0.6228 (8)	0.8392 (6)	0.0573 (6)	0.0395 (18)	
H22A	0.5567	0.9002	0.0737	0.047*	
C23	0.6348 (8)	0.7163 (6)	0.1179 (5)	0.0327 (16)	
C24	0.5265 (7)	0.7510 (6)	0.2567 (5)	0.0332 (16)	
H24A	0.5892	0.8137	0.2327	0.040*	
H24B	0.5502	0.7016	0.3266	0.040*	
C25	0.3637 (7)	0.8142 (6)	0.2457 (5)	0.0278 (15)	
C26	0.2625 (8)	0.7756 (6)	0.2077 (5)	0.0331 (16)	
H26A	0.3005	0.7137	0.1838	0.040*	
C27	0.1104 (8)	0.8208 (6)	0.2023 (5)	0.0359 (17)	
C28	0.0080 (9)	0.7699 (7)	0.1649 (6)	0.0450 (19)	
H28A	0.0638	0.7333	0.1208	0.054*	
H28B	−0.0750	0.8367	0.1272	0.054*	
C29	0.4177 (12)	0.4097 (10)	0.1362 (8)	0.081 (3)	
H29A	0.4380	0.3623	0.0948	0.097*	
H29B	0.5156	0.4160	0.1553	0.097*	
Cl1	0.3250 (4)	0.3302 (3)	0.2405 (2)	0.0965 (10)	
Cl2	0.3166 (4)	0.5569 (3)	0.0682 (2)	0.0954 (10)	

### Atomic displacement parameters (Å^2^)

**Table:**

	*U*^11^	*U*^22^	*U*^33^	*U*^12^	*U*^13^	*U*^23^
Br1	0.0579 (7)	0.0656 (7)	0.0897 (8)	0.0107 (5)	−0.0290 (5)	−0.0257 (6)
Br2	0.0413 (5)	0.0500 (5)	0.0638 (6)	−0.0137 (4)	−0.0119 (4)	−0.0123 (4)
O1	0.031 (3)	0.026 (2)	0.033 (3)	−0.008 (2)	−0.007 (2)	−0.006 (2)
O2	0.034 (3)	0.026 (2)	0.044 (3)	−0.005 (2)	−0.009 (2)	−0.011 (2)
O3	0.051 (3)	0.025 (2)	0.033 (3)	−0.011 (2)	−0.003 (2)	−0.005 (2)
O4	0.041 (3)	0.039 (3)	0.044 (3)	0.000 (2)	−0.003 (2)	−0.017 (3)
O5	0.039 (3)	0.026 (3)	0.044 (3)	−0.004 (2)	0.006 (2)	−0.011 (2)
O6	0.033 (3)	0.029 (3)	0.042 (3)	−0.008 (2)	0.011 (2)	−0.015 (2)
C1	0.027 (4)	0.066 (6)	0.055 (5)	−0.005 (4)	−0.001 (4)	−0.026 (5)
C2	0.017 (3)	0.028 (4)	0.037 (4)	0.005 (3)	−0.001 (3)	−0.006 (3)
C3	0.029 (4)	0.023 (3)	0.035 (4)	−0.002 (3)	−0.005 (3)	−0.005 (3)
C4	0.027 (4)	0.021 (3)	0.031 (4)	−0.004 (3)	0.001 (3)	−0.005 (3)
C5	0.026 (4)	0.028 (4)	0.038 (4)	−0.003 (3)	−0.002 (3)	−0.014 (3)
C6	0.028 (4)	0.035 (4)	0.033 (4)	−0.013 (3)	0.004 (3)	−0.015 (3)
C7	0.028 (4)	0.031 (4)	0.045 (5)	−0.001 (3)	−0.003 (3)	−0.015 (3)
C8	0.040 (4)	0.029 (4)	0.049 (5)	−0.011 (3)	−0.002 (4)	−0.019 (4)
C9	0.038 (4)	0.042 (4)	0.036 (4)	−0.015 (3)	−0.009 (3)	−0.012 (4)
C10	0.028 (4)	0.044 (4)	0.036 (4)	−0.007 (3)	−0.003 (3)	−0.015 (4)
C11	0.024 (4)	0.030 (4)	0.032 (4)	−0.008 (3)	−0.001 (3)	−0.010 (3)
C12	0.042 (4)	0.026 (4)	0.039 (4)	0.000 (3)	−0.005 (3)	−0.015 (3)
C13	0.033 (4)	0.029 (4)	0.038 (5)	0.002 (3)	−0.019 (3)	−0.003 (3)
C14	0.035 (4)	0.029 (4)	0.043 (5)	−0.011 (3)	0.000 (3)	−0.014 (3)
C15	0.037 (4)	0.032 (4)	0.043 (5)	−0.008 (3)	−0.003 (3)	−0.012 (3)
C16	0.046 (5)	0.028 (4)	0.051 (5)	0.007 (3)	−0.007 (4)	−0.015 (4)
C17	0.032 (4)	0.044 (5)	0.055 (5)	0.008 (3)	−0.001 (4)	−0.022 (4)
C18	0.025 (4)	0.043 (4)	0.037 (4)	−0.007 (3)	0.001 (3)	−0.023 (4)
C19	0.057 (5)	0.042 (5)	0.052 (6)	−0.013 (4)	0.017 (4)	−0.025 (4)
C20	0.062 (6)	0.048 (5)	0.036 (5)	−0.023 (4)	0.009 (4)	−0.009 (4)
C21	0.053 (5)	0.034 (4)	0.039 (5)	−0.005 (4)	−0.002 (4)	−0.003 (4)
C22	0.026 (4)	0.034 (4)	0.048 (5)	0.004 (3)	−0.004 (3)	−0.010 (4)
C23	0.036 (4)	0.034 (4)	0.032 (4)	−0.012 (3)	0.007 (3)	−0.016 (3)
C24	0.030 (4)	0.035 (4)	0.035 (4)	−0.009 (3)	0.009 (3)	−0.015 (3)
C25	0.020 (3)	0.026 (3)	0.035 (4)	−0.008 (3)	0.002 (3)	−0.009 (3)
C26	0.034 (4)	0.025 (3)	0.031 (4)	−0.001 (3)	0.008 (3)	−0.007 (3)
C27	0.026 (4)	0.040 (4)	0.032 (4)	−0.004 (3)	0.002 (3)	−0.006 (3)
C28	0.040 (5)	0.047 (5)	0.051 (5)	−0.013 (4)	−0.001 (4)	−0.019 (4)
C29	0.055 (7)	0.092 (8)	0.084 (8)	−0.008 (6)	−0.004 (6)	−0.027 (7)
Cl1	0.100 (2)	0.122 (3)	0.075 (2)	−0.042 (2)	0.0117 (17)	−0.0399 (19)
Cl2	0.116 (3)	0.089 (2)	0.092 (2)	−0.0152 (19)	−0.0132 (19)	−0.0474 (18)

### Geometric parameters (Å, º)

**Table:**

Br1—C1	1.960 (8)	C12—H12B	0.9900
Br2—C28	1.971 (8)	C13—H13A	0.9900
O1—C6	1.358 (8)	C13—H13B	0.9900
O1—C5	1.419 (8)	C14—C15	1.484 (10)
O2—C11	1.376 (8)	C14—H14A	0.9900
O2—C12	1.419 (8)	C14—H14B	0.9900
O3—C13	1.412 (8)	C15—H15A	0.9900
O3—C14	1.424 (8)	C15—H15B	0.9900
O4—C16	1.393 (9)	C16—C17	1.507 (11)
O4—C15	1.423 (9)	C16—H16A	0.9900
O5—C18	1.384 (8)	C16—H16B	0.9900
O5—C17	1.431 (8)	C17—H17A	0.9900
O6—C23	1.360 (8)	C17—H17B	0.9900
O6—C24	1.438 (8)	C18—C19	1.367 (10)
C1—C2	1.508 (9)	C18—C23	1.405 (9)
C1—H1A	0.9900	C19—C20	1.392 (10)
C1—H1B	0.9900	C19—H19A	0.9500
C2—C3	1.363 (9)	C20—C21	1.377 (11)
C2—C27	1.414 (10)	C20—H20A	0.9500
C3—C4	1.394 (9)	C21—C22	1.375 (10)
C3—H3A	0.9500	C21—H21A	0.9500
C4—C25	1.399 (9)	C22—C23	1.387 (9)
C4—C5	1.499 (9)	C22—H22A	0.9500
C5—H5A	0.9900	C24—C25	1.502 (9)
C5—H5B	0.9900	C24—H24A	0.9900
C6—C7	1.397 (9)	C24—H24B	0.9900
C6—C11	1.402 (9)	C25—C26	1.379 (9)
C7—C8	1.386 (10)	C26—C27	1.371 (9)
C7—H7A	0.9500	C26—H26A	0.9500
C8—C9	1.355 (10)	C27—C28	1.485 (10)
C8—H8A	0.9500	C28—H28A	0.9900
C9—C10	1.386 (10)	C28—H28B	0.9900
C9—H9A	0.9500	C29—Cl1	1.741 (11)
C10—C11	1.375 (9)	C29—Cl2	1.746 (11)
C10—H10A	0.9500	C29—H29A	0.9900
C12—C13	1.494 (9)	C29—H29B	0.9900
C12—H12A	0.9900		
			
C6—O1—C5	118.4 (5)	O4—C15—C14	109.3 (6)
C11—O2—C12	116.1 (5)	O4—C15—H15A	109.8
C13—O3—C14	110.7 (5)	C14—C15—H15A	109.8
C16—O4—C15	114.6 (6)	O4—C15—H15B	109.8
C18—O5—C17	116.6 (5)	C14—C15—H15B	109.8
C23—O6—C24	117.1 (5)	H15A—C15—H15B	108.3
C2—C1—Br1	113.0 (5)	O4—C16—C17	114.4 (6)
C2—C1—H1A	109.0	O4—C16—H16A	108.7
Br1—C1—H1A	109.0	C17—C16—H16A	108.7
C2—C1—H1B	109.0	O4—C16—H16B	108.7
Br1—C1—H1B	109.0	C17—C16—H16B	108.7
H1A—C1—H1B	107.8	H16A—C16—H16B	107.6
C3—C2—C27	119.0 (6)	O5—C17—C16	107.8 (6)
C3—C2—C1	119.0 (7)	O5—C17—H17A	110.2
C27—C2—C1	121.9 (7)	C16—C17—H17A	110.2
C2—C3—C4	122.8 (6)	O5—C17—H17B	110.2
C2—C3—H3A	118.6	C16—C17—H17B	110.2
C4—C3—H3A	118.6	H17A—C17—H17B	108.5
C3—C4—C25	118.5 (6)	C19—C18—O5	124.0 (6)
C3—C4—C5	120.0 (6)	C19—C18—C23	120.6 (7)
C25—C4—C5	121.4 (6)	O5—C18—C23	115.4 (6)
O1—C5—C4	108.5 (5)	C18—C19—C20	119.7 (7)
O1—C5—H5A	110.0	C18—C19—H19A	120.1
C4—C5—H5A	110.0	C20—C19—H19A	120.1
O1—C5—H5B	110.0	C21—C20—C19	120.2 (7)
C4—C5—H5B	110.0	C21—C20—H20A	119.9
H5A—C5—H5B	108.4	C19—C20—H20A	119.9
O1—C6—C7	125.0 (6)	C22—C21—C20	120.1 (7)
O1—C6—C11	116.3 (6)	C22—C21—H21A	119.9
C7—C6—C11	118.7 (6)	C20—C21—H21A	119.9
C8—C7—C6	119.7 (7)	C21—C22—C23	120.6 (7)
C8—C7—H7A	120.2	C21—C22—H22A	119.7
C6—C7—H7A	120.2	C23—C22—H22A	119.7
C9—C8—C7	120.4 (6)	O6—C23—C22	125.5 (6)
C9—C8—H8A	119.8	O6—C23—C18	115.8 (6)
C7—C8—H8A	119.8	C22—C23—C18	118.7 (7)
C8—C9—C10	121.5 (7)	O6—C24—C25	112.6 (6)
C8—C9—H9A	119.2	O6—C24—H24A	109.1
C10—C9—H9A	119.2	C25—C24—H24A	109.1
C11—C10—C9	118.7 (7)	O6—C24—H24B	109.1
C11—C10—H10A	120.6	C25—C24—H24B	109.1
C9—C10—H10A	120.6	H24A—C24—H24B	107.8
C10—C11—O2	125.0 (6)	C26—C25—C4	117.8 (6)
C10—C11—C6	121.0 (6)	C26—C25—C24	120.2 (6)
O2—C11—C6	114.0 (6)	C4—C25—C24	121.8 (6)
O2—C12—C13	107.9 (6)	C27—C26—C25	124.3 (7)
O2—C12—H12A	110.1	C27—C26—H26A	117.9
C13—C12—H12A	110.1	C25—C26—H26A	117.9
O2—C12—H12B	110.1	C26—C27—C2	117.5 (7)
C13—C12—H12B	110.1	C26—C27—C28	120.9 (7)
H12A—C12—H12B	108.4	C2—C27—C28	121.5 (6)
O3—C13—C12	109.9 (6)	C27—C28—Br2	110.5 (5)
O3—C13—H13A	109.7	C27—C28—H28A	109.6
C12—C13—H13A	109.7	Br2—C28—H28A	109.6
O3—C13—H13B	109.7	C27—C28—H28B	109.6
C12—C13—H13B	109.7	Br2—C28—H28B	109.6
H13A—C13—H13B	108.2	H28A—C28—H28B	108.1
O3—C14—C15	109.6 (6)	Cl1—C29—Cl2	113.4 (6)
O3—C14—H14A	109.7	Cl1—C29—H29A	108.9
C15—C14—H14A	109.7	Cl2—C29—H29A	108.9
O3—C14—H14B	109.7	Cl1—C29—H29B	108.9
C15—C14—H14B	109.7	Cl2—C29—H29B	108.9
H14A—C14—H14B	108.2	H29A—C29—H29B	107.7
			
C4—C5—O1—C6	169.4 (5)	O4—C16—C17—O5	−65.8 (9)
C25—C24—O6—C23	101.4 (7)	C17—O5—C18—C19	35.0 (10)
Br1—C1—C2—C3	102.2 (7)	C17—O5—C18—C23	−144.6 (7)
Br1—C1—C2—C27	−81.3 (8)	O5—C18—C19—C20	179.4 (7)
C27—C2—C3—C4	−0.5 (10)	C23—C18—C19—C20	−1.0 (12)
C1—C2—C3—C4	176.1 (6)	C18—C19—C20—C21	0.2 (13)
C2—C3—C4—C25	−0.2 (10)	C19—C20—C21—C22	−1.4 (13)
C2—C3—C4—C5	177.8 (6)	C20—C21—C22—C23	3.5 (12)
C6—O1—C5—C4	169.4 (5)	C24—O6—C23—C22	−27.0 (10)
C3—C4—C5—O1	123.9 (6)	C24—O6—C23—C18	153.7 (6)
C25—C4—C5—O1	−58.2 (8)	C21—C22—C23—O6	176.5 (7)
C5—O1—C6—C7	21.1 (9)	C21—C22—C23—C18	−4.3 (11)
C5—O1—C6—C11	−160.9 (6)	C19—C18—C23—O6	−177.6 (7)
O1—C6—C7—C8	178.4 (7)	O5—C18—C23—O6	1.9 (9)
C11—C6—C7—C8	0.5 (10)	C19—C18—C23—C22	3.1 (11)
C6—C7—C8—C9	−0.1 (11)	O5—C18—C23—C22	−177.4 (6)
C7—C8—C9—C10	0.2 (11)	C23—O6—C24—C25	101.4 (7)
C8—C9—C10—C11	−0.7 (11)	C3—C4—C25—C26	1.1 (9)
C9—C10—C11—O2	−179.0 (6)	C5—C4—C25—C26	−176.9 (6)
C9—C10—C11—C6	1.1 (10)	C3—C4—C25—C24	−174.8 (6)
C12—O2—C11—C10	−1.8 (10)	C5—C4—C25—C24	7.3 (10)
C12—O2—C11—C6	178.1 (6)	O6—C24—C25—C26	13.4 (9)
O1—C6—C11—C10	−179.1 (6)	O6—C24—C25—C4	−170.8 (6)
C7—C6—C11—C10	−1.0 (10)	C4—C25—C26—C27	−1.5 (10)
O1—C6—C11—O2	1.0 (9)	C24—C25—C26—C27	174.4 (6)
C7—C6—C11—O2	179.1 (6)	C25—C26—C27—C2	0.8 (10)
C11—O2—C12—C13	178.5 (6)	C25—C26—C27—C28	−176.9 (7)
C14—O3—C13—C12	−179.6 (6)	C3—C2—C27—C26	0.2 (10)
O2—C12—C13—O3	68.1 (7)	C1—C2—C27—C26	−176.4 (7)
C13—O3—C14—C15	177.8 (6)	C3—C2—C27—C28	177.9 (6)
C16—O4—C15—C14	174.1 (5)	C1—C2—C27—C28	1.4 (11)
O3—C14—C15—O4	76.4 (7)	C26—C27—C28—Br2	95.5 (7)
C15—O4—C16—C17	87.4 (8)	C2—C27—C28—Br2	−82.2 (8)
C18—O5—C17—C16	166.6 (6)		

### Hydrogen-bond geometry (Å, º)

**Table:**

*D*—H···*A*	*D*—H	H···*A*	*D*···*A*	*D*—H···*A*
C15—H15*B*···O5	0.99	2.38	2.996 (9)	120
C29—H29*B*···O5	0.99	2.42	3.352 (13)	158
